# Complementary and alternative therapies for poststroke depression: A protocol for systematic review and network meta-analysis

**DOI:** 10.1097/MD.0000000000021995

**Published:** 2020-09-18

**Authors:** Kai Song, Fanjie Xiong, Yating Wang, Haiyan Wang, Ailing Huang, Hong Zhang

**Affiliations:** a College of acupuncture and Tuina; b College of Clinical Medicine, Chengdu University of Traditional Chinese Medicine, Chengdu, Sichuan Province; c Department of Acupuncture and Moxibustion, Affiliated hospital of Gansu university of traditional Chinese medicine, Lanzhou, Gansu Province, China.

**Keywords:** complementary and alternative therapies, poststroke depression, protocol, systematic review

## Abstract

**Background:**

Poststroke depression (PSD) is an important complication of stroke, resulting in increased disability and mortality, which is a great threat to stroke survivors and public health. Complementary and alternative medicine (CAM) therapies is widely used in the treatment of PSD, However, the selection strategies of different CAM approaches in clinical practice is still not clear, and the purpose of this protocol is to compare the efficacy and acceptability of different CAM therapies using systematic review and network meta-analysis.

**Methods:**

According to the strategy, the authors will retrieve a total of seven electronic databases by August 2020, including PubMed, the Cochrane Library, EMbase, China National Knowledge Infrastructure, China Biological Medicine, Chinese Scientific Journals Database, and Wan-fang databases. The network meta-analysis will be performed using Aggregate Data Drug Information System 1.16.8 and Stata 13.0 software. In addition, the Cochrane Collaboration's tool is employed for the methodological quality, and the quality of evidence will be evaluated according to the Grading of Recommendations Assessment, Development, and Evaluation system.

**Results:**

This study will provide a reliable evidence for the selection strategy of CAM therapies for PSD.

**Conclusion:**

The results of this study will provide references for evaluating the effects of different CAM therapies on PSD, and provide decision-making references for clinical practitioners, patients, and health policy makers.

**Ethics and dissemination:**

This study does not require ethical approval. the results will be disseminated through a peer-reviewed publication.

**OSF registration number:**

DOI 10.17605/OSF.IO/TNGH6.

## 1 Introduction

Poststroke depression (PSD) is the most common neuropsychiatric consequences of stroke,^[1]^ occurring in 29% to 33% of stroke survivors.^[2,3]^ It is estimated that nearly 2 million individuals in the United States are dealing with PSD at any given time.^[4]^ The major symptoms of early PSD (within the first 3 months after stroke) are dysphoria, melancholia, and vegetative signs.^[5,6]^ The current evidence indicates that the neurobiological factors may be the main factors associated with PSD, specifically includes change in ascending monoamine pathways, excess of proinflammatory cytokines, dysfunction of the hypothalamic-pituitary adrenal axis and alterations in neuroplasticity.^[7]^ Studies have demonstrated that PSD can significantly compromise quality of life, including affecting cognitive function, social activity, and stroke rehabilitation. Moreover, it is also associated with increase mortality risk.^[8,9]^ Current research suggests that disability, personal and family history of a psychiatric illness, and high overall medical burden may be risk factors for PSD.^[10,11]^ Due to the complexity of diagnosis and the uncertainty of various screening tools, consequently, only a small percentage of PSD patients can be accurately diagnosed and treated.^[12]^ The main therapeutic strategies for PSD include pharmacological and nonpharmacological interventions (eg, psychotherapy, surgical therapy, electroconvulsive therapy). In the pharmacological interventions, it has been suggested that Selective Serotonin Reuptake Inhibitors is the first line treatment,^[13]^ such as fuoxetine, sertraline, and citalopram.^[14]^ There is no doubt that the pharmacological therapy for PSD has a positive effect. However, there was also a significant increase in adverse events,^[15]^ such as gastroenterological symptoms, epilepsy/ seizures and hyponatremia.^[7]^ In addition, intolerance of antidepressants by some stroke survivors, and poor treatment adherence may further reduce the impact of drugs in PSD treatment.^[16]^ Thus, better strategies for effective PSD treatment are needed.

Complementary and alternative medicine (CAM) therapies refers to a diverse range of healing techniques that are not considered established or standard practices in western medicine.^[17]^ Many CAM modalities have been used by stroke survivors all around the world,^[18]^ including acupuncture, meridian acupressure, light therapy, exercise, repetitive transcranial magnetic stimulation (rTMS), music therapy, herbal medicines and so on. One study reports that 46% of stroke survivors engage in some form of complementary medicine.^[19]^ In Korea, 54% of stroke patients used CAM therapies, and 16% who felt that it can effectively achieve psychological relaxation.^[20]^ In recent years, CAM therapies has been increasingly sought by people with PSD.^[21]^ It is reported that acupuncture is more effective than short-term use of antidepressants in patients with PSD.^[22]^ Deng et al^[23]^ found that rTMS is a beneficial therapeutic method for managing PSD and may even be superior in efficacy to selective serotonin reuptake inhibitors. Kim et al^[24]^ reported the positive roles of music therapy on improvement of depressive mood and anxiety in stroke patients. A study from Kang et al^[25]^ has proven Meridian acupressure benefits in improvement of PSD.

Despite the numerous CAM therapies for PSD has been evaluated in previous randomized controlled trials (RCTs), However, majority have not been quantitatively analyzed in head-to-head comparisons. Thus, we performed a network meta-analysis (NMA) of all RCTs involving CAM therapies for PSD, to compare and comprehensively rank all available CAM therapies, and assess efficacy and acceptability of different CAM therapies.

## 2 Methods

### 2.1 Protocol and registration

This protocol based on the Preferred Reporting Items for Systematic Reviews and Meta-Analyses Protocols (PRISMA-P) guidelines,^[26]^ and the checklist will be upload as an attachment. The NMA protocol has been registered on Open Science Framework platform (https://osf.io/tngh6/), registration number: DOI 10.17605/OSF.IO/TNGH6.

### 2.2 Ethics

This research does not require ethical approval.

### 2.3 Eligibility criteria

The inclusion and exclusion criteria of this research were based on the PICO principle (participant, intervention, comparator, outcome).

#### 2.3.1 Type of participant

All studies including patients with PSD, regardless of age, gender, ethnicity, profession, and educational background. Specific diagnostic criteria include:

1. A clinical diagnosis of ischemic or hemorrhagic stroke;
2. PSD confirmed by specific criteria, such as Diagnostic and Statistical Manual of Mental Disorders (DSM-III, DSM-IV, and DSM-V), International Classification of Diseases (ICD-10) or depression scales, such as Hamilton Depression Scale (HAMD) scale.

#### 2.3.2 Type of interventions and comparators

Interventions comprised complementary and alternative therapies for treating PSD (acupuncture, meridian acupressure, light therapy, Yoga, rTMS, music therapy, herbal medicines, Tai Chi, and so on), These interventions can be used alone or in combination. Controlled interventions included control groups with no treatment, sham/placebo groups, or other conventional therapy.

#### 2.3.3 Type of outcomes

*Primary outcomes*. The primary outcome was the change in overall depressive symptoms, measured by standardized instrument such as the Hamilton Depression Scale (HAMD)^[27]^ or Montgomery-Asberg Depression Rating Scale (MADRS)^[28]^ or Beck Depression Inventory (BDI)^[29]^ or Zung Self-Rating Depression Scale (ZDS)^[30]^ from baseline to end point.

*Secondary outcomes*

1. Activities of daily living measured by established and validated assessment tools, for example, Barthel index.^[31]^
2. Quality of life obtained from the corresponding scale, for example, the Stroke Specific Quality of Life Scale.^[32]^
3. Adverse events.

#### 2.3.4 Study design

All relevant RCTs using CAM therapies for the PSD will be included, Quasi-RCTs, duplications, animal trails, review documents, clinical experience, letters, meeting abstracts, and case reports will be excluded. Additionally, only English and Chinese literature will be search for this study.

### 2.4 Literature retrieval strategy

The following 7 databases will be searched by computer for RCTs on complementary and alternative therapies for PSD: PubMed, the Cochrane Library (issue 8, 2020), EMbase, China National Knowledge Infrastructure (CNKI), China Biological Medicine (CBM), Chinese Scientific Journals Database (VIP), and Wan-fang databases. The time limit of document retrieval is from the establishment of each database to August 31, 2020. The language is confined to English and Chinese. Moreover, inclusive literature from the field and references from previous evaluations will be manually retrieved to find other potentially relevant articles. The retrieval mode will be a combination of free words and medical subject headings terms, including: “poststroke depression,” “PSD,” “stroke,” “depression,” “complementary and alternative therapy,” “acupuncture,” “meridian acupressure,” “light therapy,” “Yoga",” “rTMS,” “music therapy”,” “herbal medicines,” and “Tai Chi,” etc. The following terms will be used in the Chinese database retrieval: “yiyu,” “cuzhong,” “zhongfeng,” “buchongtidai,” “zhenjiu,” “guangliaofa,” “caoyao,” “yujia,” “taiji,” “yinyueliaofa,” etc. The initial retrieval strategy for PubMed is shown in Table 1, which will be adjusted in accordance with specific databases.

**Table 1:**
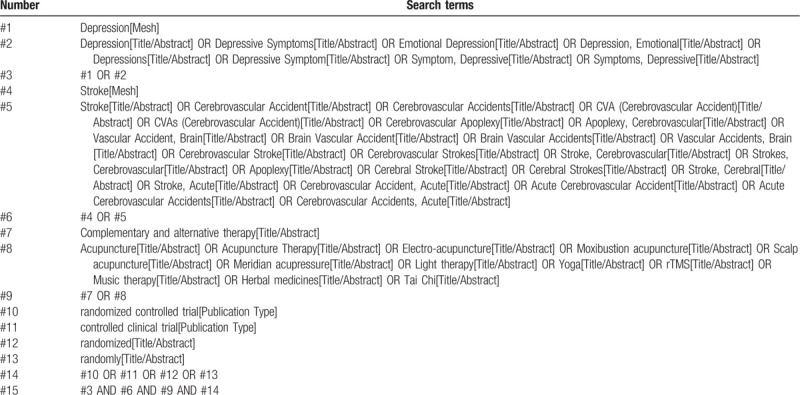
Search strategy of the PubMed.

### 2.5 Literature selection and data extraction

**Figure 1:**
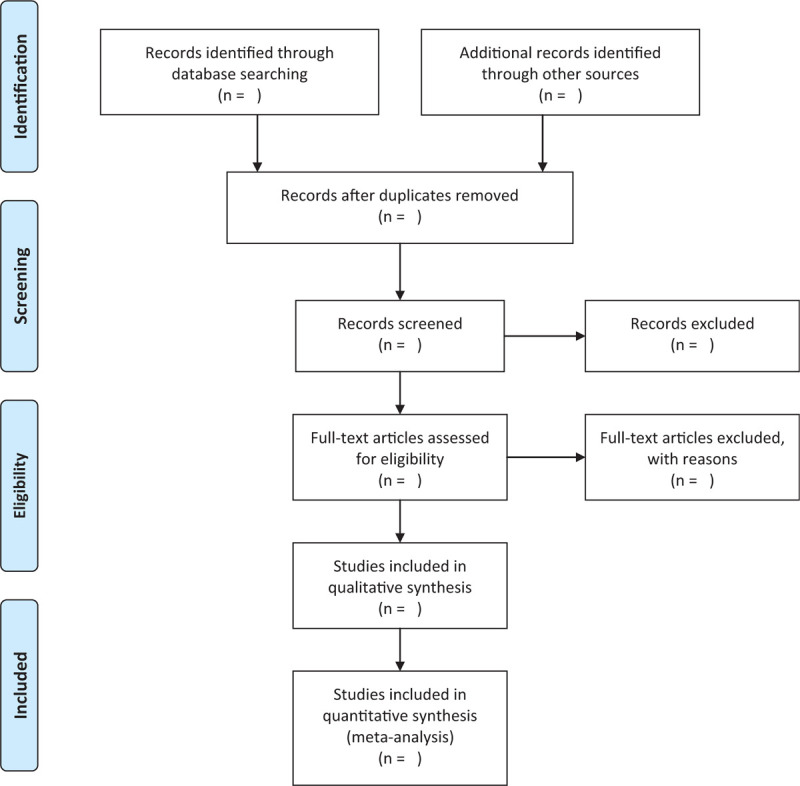
Flow chart of literature screening.

The study selection program will follow the Prisma guidelines, As shown in Figure 1, The 2 researchers (Kai Song and Fanjie Xiong) will independently screen literatures according to inclusion and exclusion criteria:

1. The retrieved literatures will be imported into Endnote X9 software for rechecking, and duplicate references are removed;
2. By reading the title and preliminarily screening the abstract, exclude the literature that obviously does not meet the inclusion criteria;
3. Download and read the full text for rescreening;
4. After the final inclusion, the predesigned data extraction table is used for data extraction, and the results will be cross-checked;
5. If there is any disagreement, the third researcher (Ailing Huang) will be asked to assist in the judgment.

The main content of data extraction includes basic information of literature (title, journal, author, country, publication year), basic situation of the research object (mean age, gender, sample size, intervention and comparator, course of treatment), outcome data (mean, standard deviation, follow-up time, and treatment-related adverse events). At the same time, the key factors of bias risk assessment are extracted.

### 2.6 Quality assessment/methodological quality of included studies

Methodological quality will be assessed based on the Cochrane Collaboration's tool (Cochrane Handbook 5.1.0). Two trained researchers (Kai Song and Ailing Huang) will independently evaluate the risk of bias of the included studies. In case of dispute, submit to corresponding author (Hong Zhang) for arbitration.

Cochrane bias risk assessment tool will be used to assess the risk of RCTs being included in NMA, including^[33]^:

1. random sequence generation;
2. allocation concealment;
3. blinding of participants and personnel;
4. blinding of outcome assessment;
5. incomplete outcome data;
6. selective reporting;
7. other bias.

### 2.7 Data synthesis and statistical methods

#### 2.7.1 Network meta-analysis

This study will use Aggregate Data Drug Information System 1.16.8 for NMA.^[34]^ Aggregate Data Drug Information System software uses markov chain-monte carlo (MCMC) algorithm to for priori evaluation and processing the extracted data based on bayesian framework, so as to provide support for further research and decision. Preset model parameters: 4 chains are used for simulation analysis, with an initial value of 2.5, a step size of 10, 20,000 annealing times, and 50,000 simulation iterations. Firstly, the network evidence plot is generated according to different outcome indicators, standardized mean differences or odds ratios is used as the effect quantity for statistical analysis, both with 95% credible intervals. According to the results of the NMA, rank probability plot of various CAM therapies is generated and sorted by dominance, with Rank1 being the optimal sort. However, if the score standard of the outcome is lower and greater, then the lowest ranked prediction sequence is the optimal sequence.

#### 2.7.2 Statistical model selection

Node-split model is used to verify the consistency of the corresponding data. If there is no statistical difference (*P* > .05) between direct comparison and indirect comparison, the consistency model is used, whereas the inconsistency model is used for analysis. If the consistency model is adopted, then the stability of the results is verified by the inconsistency model: when the inconsistency factors including 0, at the same time inconsistency standard deviation including 1 says the result of consistency model is more stable and reliable. At the same time, various analysis models are iterated with preset parameters, and the convergence of iteration effect is judged by potential scale reduced factor (PSRF). When the PSRF value is close to or equal to 1 (1 ≤ PSRF ≤ 1.05), the convergence is complete, the model has good stability, and the conclusion of analysis is reliable. If the PSRF value is not in this range, the iteration continues manually until the PSRF value reaches the range standard.

#### 2.7.3 Heterogeneity test

Before the combination of effect size, the heterogeneity of the included literature is tested using Stata 13.0 software. When inter-study heterogeneity exists, the random effect model is used. For comparison of each pair, heterogeneity is assessed by the statistic *I*^2^ value. When *I*^2^ > 50%, it indicates that there is heterogeneity between studies, and the source of heterogeneity should be further searched. When *I*^2^ < 50%, inter-study heterogeneity is considered to be small or there is no obvious heterogeneity.

#### 2.7.4 Sensitivity analysis

If necessary, the sensitivity analysis will be used to assess the effect of each study on the random effects model. The sensitivity of the general combined effect of all outcome indicators is analyzed by the exclusion method. That is, each study is excluded, and the remaining studies will be re-analyzed to identify the stability of the results. If there is no qualitative change in the combined effect showed in the results, the results are stable.

#### 2.7.5 Subgroup analysis

If necessary, we will conduct a subgroup analysis of duration of treatment, age, the clinical course of PSD, and research quality.

#### 2.7.6 Small sample effect/publication bias

If 10 or more studies are included in the NMA, a comparison-adjusted funnel plot is developed using Stata to evaluate the presence of small sample effects or publication bias in the intervention network. Descriptive analysis will be carried out through the symmetry of funnel plot. If the plot is asymmetric and there is no inverted funnel shape, it indicates that there may be publication bias. This may be related to the difficulty in the publication of the literature with negative results and the low quality of the inclusion methods.

#### 2.7.7 Dealing with missing data

If the required data is lost or incomplete, we will contact the corresponding author of the original document or the relevant email address of the first author. If there is no response, the record is excluded.

#### 2.7.8 Evaluating the quality of the evidence

To grade evidence quality and understand the current situation of evidence rating thereby analyzing possible problems, The Grading of Recommendations Assessment, Development and Evaluation instrumental will be used to assess the quality of evidence in the NMA.^[35]^ Based on the risk of bias, inconsistency, imprecision, indirection, and publication bias, Grading of Recommendations Assessment, Development and Evaluation grades evidence quality into four levels: high, medium, low, and very low.

#### 2.7.9 Patient and public involvement

There was no patient or public involvement in the preparation of this protocol.

## 3 Discussions

PSD is a common sequela of stroke, which can reduce the quality of life and increases the risk of natural and suicidal death, and bring heavy burden to patients and their families. Globally, an ever-growing number of stroke survivors with PSD using CAM therapies to treat their symptoms, The advantage of CAM therapy is that it is not only considered as effective as a conventional pharmacological interventions, but also more natural and economical, with fewer side effects and available without need of prescription.^[36]^ However, there is no decision-making conclusion as to which CAM strategy should be preferred in clinical practice of PSD. Thus, our study employed an NMA of relevant RCTs of CAM therapies for PSD, including acupuncture, meridian acupressure, light therapy, exercise, rTMS, music therapy, herbal medicines etc, to evaluate the efficacy and acceptability of different CAM therapies treatments. As we have seen, this study will be the first NMA in this respect. We hope that the study results will provide useful references for PSD clinical practice or guideline to a certain extent.

However, there are some potential limitations that are predictable in this study. For example, due to the limitations of language ability, we only search for literature in English and Chinese, which may lead to selection bias. In addition, difference of methodological quality in the trials may lead to significant heterogeneity.

Nevertheless, the ranking of different CAM therapies can guide clinical decision makers to select the best therapeutic strategies for PSD. The findings and results of this study will be published in a peer-reviewed journal.

## Author contributions

**Conceptualization:** Kai Song, Fanjie Xiong, Yating Wang.

**Data curation:** Kai Song, Fanjie Xiong, Ailing Huang.

**Formal analysis:** Fanjie Xiong, Haiyan Wang.

**Funding acquisition:** Hong Zhang.

**Methodology:** Kai Song, Fanjie Xiong, Ailing Huang.

**Project administration:** Kai Song, Yating Wang, Haiyan Wang.

**Writing – original draft**: Kai Song, Fanjie Xiong.

**Writing – review & editing:** Hong Zhang.

All the authors have approved the publication of the protocol

## Abbreviations

Abbreviations: CAM = complementary and alternative medicine, DSM = Diagnostic and Statistical Manual of Mental Disorders, NMA = Network Meta-analysis, PSD = post-stroke depression, PSRF = potential scale reduced factor, RCTs = randomized controlled trials, SSRIs = selective serotonin reuptake inhibitors.
